# The association between polymorphisms in the *MDR1* gene and risk of cancer: a systematic review and pooled analysis of 52 case–control studies

**DOI:** 10.1186/1475-2867-13-46

**Published:** 2013-05-20

**Authors:** Ling-Hui Wang, Yan-Bin Song, Wen-Ling Zheng, Ling Jiang, Wen-Li Ma

**Affiliations:** 1 Biochemistry and Molecular Biology Director, Institute of Genetic Engineering, Southern Medical University, Guangzhou, 510515, People’s Republic of China

**Keywords:** *MDR1*, Polymorphism, Cancer risk, Meta-analysis

## Abstract

**Background:**

The multidrug resistance (*MDR*) 1 gene encodes a 170-kDa membrane transporter called P-glycoprotein, which plays an important role in protecting cells against lipophilic xenobiotics by the way of an ATP-dependent cellular efflux mechanism. Three polymorphisms of *MDR1*, 3435C > T located in exon 26, 1236C > T in exon 12 and 2677G > T/A in exon 21 were the most extensively studied and were identified functionally important and ethnically diverse mapping to the gene region. Considering the potential influence of altering *MDR1* activity, it is plausible that *MDR1* polymorphisms might play a role in the development of cancer. Although the effects of *MDR1* polymorphisms on susceptibility to human cancer have been investigated in many studies, the results still remain conflicting.

**Methods:**

To resolve these conflicts, we performed a quantitative synthesis of the association between these three polymorphisms and cancer risk, including 52 studies (15789 cases and 20274 controls) for 3435C > T polymorphism, 10 studies (2101 cases and 2842 controls) for 1236C > T polymorphism and 18 studies (3585 cases and 4351 controls) for 2677G > T/A polymorphism.

**Results:**

The stratified analyses for 3435C > T polymorphism, individuals with T-allele in 3435C > T had significantly higher ALL risks (TT versus CC: OR =1.286, 95% CI =1.123-1.474); significantly elevated risks were observed among Caucasian populations (TT versus CC: OR =1.276, 95% CI =1.112-1.464). When restricting the analysis to the source of controls, we found that HB (hospital-based) genetic models had higher risks (TT versus CC: OR =1.307, 95% CI =1.046-1.632), as well as in PB (population-based) genetic models (TT versus CC: OR =1.294, 95% CI =1.079-1.55).

The T/A-allele frequency of 2677G > T/A polymorphism was associated with higher risk of cancer (TT + TA + AA vs. GG: OR =1.348, 95% CI =1.031-1.762), significantly elevated risks were observed among Asian populations (TT + TA + AA vs. GG: OR =1.642, 95% CI =1.340-2.012), and elevated risks could be associated with PB models (TT + TA + AA vs. GG: OR =1.641, 95% CI =1.018-2.646).

**Conclusions:**

Our meta-analysis suggested that 3435C > T polymorphism and 2677G > T/A polymorphism were associated with cancer risk when all studies were pooled together, while 1236C > T polymorphism not.

## Background

The human multidrug resistance 1 (*MDR1* or *ABCB1*) gene encodes a 170-kDa membrane transport protein called P-glycoprotein. For minimizing the exposure of potential toxic compounds to the cellular homeostasis, P-glycoprotein is expressed primarily in regions that act as epithelial barriers or perform excretory functions, including blood-tissue barrier, the gastrointestinal tract, liver and kidney. Therefore, P-glycoprotein can play the role of sweeper by extruding several exogenous and endogenous substances, using ATP-dependent efflux pump [1-4]. The alteration of the cellular defense mechanism mediated by P-gp has been speculated to be closely associated with the development of various cancers including hepatocarcinoma, colorectal carcinoma, acute lymphoblastic leukemia and gall bladder tumors [5-8]. These suggest that *MDR1* may play an important role in the elimination of carcinogens, and the mutation of *MDR1* may lead to human malignancies [5]. Several studies try to prove the causal function of P-gp in tumorigenesis by animal experiments. One study by Mochida Y et al. suggested that the absence of the P-gp role suppressed the development of intestinal neoplasia in Apc (Min/+) mice, and a P-gp inhibitor was found to suppress tumorigenesis in rats subsequently [9,10]. While Schinkel et al. conducted a study comparing normal *MDR1*a (+/+) mice (*MDR1*a is the mouse equivalent to the human *MDR1* gene) to constructed *MDR1*a (−/−) disrupted mice to find that the *MDR1*a (−/−)mice resulted in cumulative toxicity of the pesticide, ivermectin due to decreased extrusion of these compounds [11]. Considering these data, we can infer that genetical absence of Pg-p expression may result in more exposure to environmental xenobiotics so that increased opportunity linked to the risk of malignancy was obtained. However, the causal relationship between *MDR1* and the tumorigenesis has not been fully elucidated yet.

Recently, at least 50 single-nucleotide polymorphisms have been reported within *MDR1* gene locus [12,13]. Among the systematic screens of this gene, Hoffmeyer et al. reported significant role that a synonymous SNP played at position 3435 located in exon 26 in the P-glycoprotein function [14]. Recent studies have found that C3435T was in linkage disequilibrium with two other common SNPs, the synonymous C1236T (exon 12) and nonsynonymous triallelic G2677T/A (exon 21) [15-17].

Considering the potential influence of these SNPs, many molecular epidemiological studies were conducted to investigate the association between these SNPs and cancer risk in humans. However, the results from different studies are to some extent divergent, but nevertheless intriguing, which may be owing to limitations in individual studies. To clarify this issue, we performed a meta-analysis with subgroup analysis from all eligible studies focusing on 3435C > T, 1236C > T and 2677G > T/A, to obtain a more precise estimation of the relationship between polymorphisms and cancer risk.

## Material and methods

### Identification and eligibility of relevant studies

All case–control studies on the association between *MDR1* polymorphisms and cancer risk published up to November 30, 2012 were identified through comprehensive searches using the PubMed and Medline database, ScienceDirect database, Springerlink database, Wiley Online Library, BioMed Central, Nature Series, Science Online, Cell Press Journals, CNKI, WanFang database with the following terms and keywords: “*MDR1*,” “*ABCB1*,” “single-nucleotide polymorphism,” and in combination with “leukemia,” “cancer,” “tumor” and “carcinoma.” The search was limited to human studies. In addition, we have especially reviewed the references cited in checked articles and identified some additional articles missed by the searching.

### Inclusion criteria

The following criteria were used for the study selection: (1) a case–control study evaluating at least one of these three polymorphisms (3435C > T, 1236C > T and 2677G > T/A) and cancer risk; (2) studies with full-text articles; (3) no overlapping data. and (4) sufficient data for estimating an odds ratio (OR) with 95% confidence interval (95% CI).

### Data extraction

Information was carefully extracted from all the eligible publications. The following data were collected from each study: first author’s name, publication date, country, ethnicity, cancer type, source of controls (population-based [PB] or hospital-based [HB] controls), genotyping method, total numbers of cases and controls and number of cases and controls for each *MDR1* polymorphism. Meanwhile, different case–control groups in one study were considered as independent studies. For each study, we did not define a minimum number of patients for inclusion in our meta-analysis.

### Statistical methods

The strength of association between *MDR1* polymorphisms and cancer risk was measured by ORs with 95% CIs. The risks (ORs) of cancer associated with the three

polymorphisms were estimated for each study. In our study, the C- allele, C-allele and G-allele were considered the reference genotypes, respectively in 3435C > T, 1236C > T and 2677G > T/A. The pooled ORs were performed for co-dominant model (TT vs. CC and TC vs. CC, TT + TA + AA vs. GG and GT + GA vs. GG), dominant model (TC + TT vs. CC, TT + TA + AA + GT + GA vs. GG) and recessive model (TT vs. TC + CC, TT + TA + AA vs. GT + GA + AA), respectively. Heterogeneity assumption was checked by a *χ*2-based Q-test. A p-value of >0.05 for the Q-test indicated a lack of heterogeneity among studies, so that the pooled OR estimate of each study was calculated by the fixed-effects model (the Mantel–Haenszel method). Otherwise, the random-effects model (the DerSimonian and Laird method) was used. Heterogeneity was quantified with the I^2^ metric, which is independent of the number of studies in the meta-analysis (I^2^ < 25%: no heterogeneity; I^2^ = 25–50%: moderate heterogeneity; I^2^ = 50–75%: large heterogeneity, I^2^ > 75%: extreme heterogeneity). Subgroup analyses were performed by cancer type (if one cancer type contained fewer than three individual studies, it was combined into an “other cancers” group), ethnicity and source of controls. Before analysis for each study, we examined whether the genotype distribution of controls was consistent with Hardy– Weinberg equilibrium using the *χ*2 test. We performed One-way sensitivity analysis by deleting a single study in the meta-analysis each time to reflect the influence of the individual data set to the pooled OR to assess the stability of the results. An estimate of potential publication bias was carried out by the funnel plot, in which the standard error of log (OR) of each study was plotted against its log (OR). An asymmetric plot suggests a possible publication bias. Funnel plot asymmetry was assessed by the method of Egger’s linear regression test, a linear regression approach to measure funnel plot asymmetry on the natural logarithm scale of the OR. The significance of the intercept was determined by the *t*-test suggested by Egger (p < 0.05 was considered a significant publication bias). All of the statistical tests used in our meta-analysis were performed by SPSS version 13.0 and STATA version 11.0 (Stata, College Station, TX).

## Results

### Eligible studies and meta-analysis databases

A total of 48 publications with 52 case–control studies exploring the association between *MDR1* 3435C > T, 1236C > T and 2677G > T/A polymorphisms and cancer risk were found [2,5,18-62]. Hence, as summarized in Table 1, 52 studies (15789 cases and 20274 controls) for 3435C > T polymorphism, 10 studies (2101 cases and 2842 controls) for 1236C > T polymorphism and 18 studies (3585 cases and 4351 controls) for 2677G > T/A polymorphism were selected in the meta-analysis, of which one publications had three independent studies and were considered separately. As summarized in Table 1, there were 25 hospital-based studies and 26 population-based studies. There were 8 acute lymphoblastic leukemia (ALL) studies, 14 colorectal cancer studies, 9 breast cancer studies, 4 gastric cancer studies, 3 renal cell cancer studies, 2 acute myelocytic leukemia (AML) studies, 2 lung cancer study, 2 B-cell chronic lymphocytic leukemia (CLL) studies, one endometrial cancer study, one esophageal squamous cell carcinoma study, one glioma study, one upper aerodigestive tract (UADT) cancers study, one multiple myeloma study, one leukemia study, one plasma cell myeloma study and one study with Hodgkin’s lymphoma (HL). Thirty-three studies were conducted in Europeans, seventeen studies were conducted in Asians. Two of the remained studies were conducted in Americans, and the other was in Mexicans. These studies indicate that the distribution of genotypes in controls was consistent with Hardy–Weinberg equilibrium. And the subjects of controls were matched for age and gender. Most of the cases were confirmed histologically or pathologically.

**Table 1:** Characteristics of studies included in the meta-analysis

**References**	**First author’s name**	**Year of publication**	**Country of origin**	**Cancer type**	**Ethnicity**	**SNPs**	**Source of control groups**	**Matching criteria**	**Genotyping methods**	**Case**	**control**	**MAF**	**HWE**
18	Rong-rong Liu	2008	Hunan, China	ALL	Asian	C3435T,C1236T,G2677T,G2677A	HB	Age, gender, weight	PCR-RFLP	48	100	0.17;0.24;0.04	0.60;0.21;0.07
19	Zhi-zhuo Du	2010	Suzhou, China	ALL	Asian	C3435T,C1236T,G2677T/A	HB	Age, gender	SNP-shot	176	170	0.30;0.38;0.27	0.00;0.28;0.23
2	Kevin Y. Urayama	2007	Northern and Central California, USA	ALL		C3435T,C1236T,G2677T/A	HB	Age, gender, Hispanic status	PCR-SBEP	294	369	0.25;0.21;0.18	0.30;0.13;0.18
3	Hiroyoshi Hattori	2007	Japan	ALL	Asian	C3435T	HB	Age, gender	Taqman	622	96	0.18	0.3
20	Jamroziak K	2004	Poland	ALL	Caucasian	C3435T	PB	Age, gender,	PCR-RELP	113	175	0.24	0
21	Evelia Leal-Ugarte	2008	Mexico	ALL		C3435T	PB	Gender	PCR-RELP	107	111	0.28	0.02
22	A’ gnes F. Semsei	2008	Hungary	ALL	Caucasian	C3435T,G2677T/A	PB	Age, gender, risk group	PCR-RELP	378	189	0.29;0.28	0.07;0.13
23	Vibeke Andersen	2009	Danish	colorectal cancer	Caucasian	C3435T,G-rs3789244-A	PB	Age, Gender,	TaqMan	359	765	0.33	0.47
24	Daniele Campa	2012	Czech Republic	colorectal cancer	Caucasian	C3435T	HB	Age, Gender,	Taqman	699	622	0.29	0.79
24	Daniele Campa	2012	Southwest Germany	colorectal cancer	Caucasian	C3435T	HB	Age, Gender, county of residence	Taqman	1809	1853	0.28	0.91
24	Daniele Campa	2012	Southwest Germany	colorectal cancer	Caucasian	C3435T	PB	Age, Gender,	Taqman	2169	1634	0.29	0.62
25	Urosˇ Potocnik	2008	Slovenia	MSI-H colorectal cancer	Caucasian	C3435T,C1236T,G2677T	HB	Age, Gender,	TaqMan	38	355	0.30;0.18;0.19	0.66;0.33;0.24
26	Nizar M Mhaidat	2011	Jordan	HL	Asian	C3435T	HB	Age	PCR-RFLP	130	120	0.35	0.11
27	Sun-Young Bae	2006	Korea	colorectal cancer	Asian	C3435T	HB	Age, Gender,	PCR-RFLP	111	93	0.16	0.01
28	Azam Khedri	2011	Iran	colorectal cancer	Caucasian	C3435T	HB	Age, Gender,	PCR-RFLP	118	137	0.35	0.56
29	Mateusz Kurzawski	2005	Poland	colorectal cancer	Caucasian	C3435T	HB	Age, Gender,	PCR-RFLP	184	188	0.29	0.27
30	Chiko Komoto	2006	Japan	colorectal cancer	Asian	C3435T,C1236T,G2677T	HB	Age, Gender,	TaqMan	48	154	0.16;0.375;0.36	0.62;0.36;0.37
30	Chiko Komoto	2006	Japan	esophageal squamous cell carcinoma	Asian	C3435T,C1236T,G2677T	HB	Age, Gender,	TaqMan	47	154	0.17;0.39;0.34	0.66;0.34;0.64
31	Bo-In Lee	2006	Korea	colorectal cancer	Asian	C3435T	HB	Age, Gender,	PCR-RFLP	64	64	0.13	0.15
32	Elena Osswald	2006	Russia	colorectal cancer	Caucasian	C3435T,G2677T/A	HB	Age, Gender, Smoking intensity	PCR-RFLP	285	275	0.26;0.20	0.35;0.42
33	Mariusz Panczyk	2009	Poland	colorectal cancer	Caucasian	C3435T,C1236T,G2677T	HB	Age, Gender,	PCR-RFLP	95	95	0.20;0.17;0.18	0.99;0.72;0.60
34	Darinka Todorova Petrova	2008	Bulgaria	colorectal cancer	Caucasian	C3435T,G2677T	HB	Age, Gender,	RT-PCR	146	160	0.25;0.21	0.73;0.32
35	J Sainz	2011	South Germany	colorectal cancer	Caucasian	C3435T	PB	Age, Gender,	PCR	1765	1784	0.27	0.6
36	Cizmarikova	2010	Eastern Slovakia	breast cancer	Caucasian	C3435T	PB	Age, Gender,	PCR-RFLP	221	113	0.27	0.6
37	M. Taheri	2010	Iran	breast cancer	Caucasian	C3435T	HB	Age, Gender,	PCR-RFLP	54	50	0.26	0.61
38	Tatari F	2009	North east of Iran	breast cancer	Caucasian	C3435T	HB	Gender,	PCR	106	77	0.29	0.59
39	Joseph George	2009	India	breast cancer	Asian	C3435T	HB	Gender,	PCR	86	68	0.15	0.7
40	Henriquez-Hernandez	2009	Spain	breast cancer	Caucasian	C3435T	HB	Gender,	PCR-RFLP	135	301	0.19	0.66
41	Nordgard	2007	Norway	breast cancer	Caucasian	C3435T,C1236T,G2677T/A	N.A.	Age, Gender,	PCR-RFLP	109	93	0.13;0.26;0.26	0.27;0.16;0.03
42	Sebahat Turgut	2007	Turkey	breast cancer	Caucasian	C3435T	PB	Gender,	PCR-RFLP	57	50	0.24	0.62
43	Wu H	2012	China	breast cancer	Asian	C3435T,C1236T,G2677T/A	PB	Age, Gender,	PCR-RFLP	1173	1244	0.17;0.41;0.17	0.01;0.04;0.94
44	Z Sabahi	2010	Iran	Gastric cancer	Caucasian	C3435T	PB	Age, Gender,	PCR	48	131	0.3	0.58
45	Mitsushige Sugimoto	2008	Japan	Gastric cancer	Asian	C3435T	HB	Age, Gender,	PCR-RFLP	150	168	0.17	0.68
46	Tomomitsu Tahara	2006	Japan	Gastric cancer	Asian	C3435T	HB	Age, Gender,	PCR-RFLP	157	104	0.21	0.64
47	P.M. MROZIKIEWICZ	2007	Poland	endometrial cancer	Caucasian	C3435T	PB	Age, Gender,	PCR	198	488	0.26	0.61
48	Federica Gemignani	2007	six Central and Eastern European countries	lung cancer	Caucasian	C3435T,G2677T/A	HB	Age, Gender,	TaqMan	299	317	0.24;0.21	0.62;0.01
49	S Haenisch	2007	Germany	renal cell cancer	Caucasian	C3435T,G2677T/A	PB	Age, Gender,	PCR-RFLP	82	164	0.31;0.22	0.58;0.14
50	Krzysztof Jamroziak	2006	Poland	AML	Caucasian	C3435T	PB	Age, Gender,	PCR-RFLP	180	180	0.23	0.64
51	Katie L. Miller	2005	USA	Glioma	Caucasian	C3435T	PB	Age, Gender,	PCR-RFLP	382	464	0.25	0.61
52	Krzysztof Jamroziak	2006	Poland	B-cell CLL	Caucasian	C3435T	PB	Age, Gender,	PCR-RFLP	110	201	0.2	0.65
53	Michael Siegsmund	2002	Germany	clear cell renal cell carcinoma (CCRCC)	Caucasian	C3435T	PB	Age, Gender,	PCR-RFLP	179	150	0.29	0.59
53	Michael Siegsmund	2002	Germany	non-CCRCC	Caucasian	C3435T	PB	Age, Gender,	PCR-RFLP	83	150	0.3	0.58
54	Guillermo Gervasini	2006	Spain	lung cancer	Caucasian	C3435T,G2677T/A	PB	Age, Gender,	TaqMan	96	86	0.26;0.31	0.61;0,。60
55	Soya Sisy Sam	2007	India	upper aerodigestive tract (UADT) cancers	Asian	C3435T	HB	Age, Gender,	PCR-RFLP	219	210	0.35	0.55
56	Krzysztof Jamroziak	2008	Poland	multiple myeloma	Caucasian	C3435T,C1236T,G2677T/A	PB	Age, Gender,	PCR-RFLP	111	96	0.19;0.15;0.18	0.66;0.01;0.55
57	V Rocha	2008	France	leukemia	Caucasian	C3435T	PB	Age, Gender,	PCR-RFLP	107	107	0.2	0.65
58	STEPHEN DRAIN	2009	UK	plasma cell myeloma	Caucasian	C3435T	PB	Age, Gender,	PCR-RFLP	92	92	0.28	0.6
59	G. Penna	2010	Italy	B-cell CLL	Caucasian	C3435T,G2677T	PB	Age, Gender,	PCR-RFLP	125	125	0.25;0.30	0.62;0.00
60	The MARIE-GENICA Consortium	2010	Germany	breast cancer	Caucasian	C3435T	PB	Age, Gender,	MALDI-TOFMS	3148	5486	0.22	0.64
61	HYUN CHANG	2009	Korea	Gastric cancer	Asian	C3435T,G2677T/A	PB	Age, Gender,	PCR	43	118	0.34;0.23	0.56;0.21
62	D Nageswara Rao	2010	India	AML	Asian	C3435T	PB	Age, Gender,	PCR-RFLP	143	249	0.28	0.6
62	D Nageswara Rao	2010	India	ALL	Asian	C3435T	PB	Age, Gender,	PCR-RFLP	147	249	0.37	0.54

Abbreviations: *HB*: hospital-based; *PB*: population-based; *PCR-RFLP*: polymerase chain reaction-restriction fragment length polymorphism; *MAF*: minor allele frequency; *APEX*: arrayed primer extensions.

### Quantitative synthesis

There was a wide variation in the T-allele and T/A-allele frequency of 3435C > T, 1236C > T and 2677G > T/A polymorphism between the two major ethnicities. For Asians, the T- allele frequency of 3435C > T was 25.52% (95% CI =23.84–27.21%), which was significantly higher than that in Caucasians (24.28%, 95% CI = 20.54–28.03%, p < 0.001). There was no statistical difference for the T- allele frequency of 1236C > T between Asians (24.28%, 95% CI = 11.37–26.56%) and Caucasians (36.11%, 95% CI = 27.74–44.47%, p =0.633). And for Asians (23.47%, 95% CI = 19.78–25.96%) whose T/A-allele frequency of 2677G > T/A polymorphism was not equivalent as Caucasians (22.87%, 95% CI = 11.12–35.82%, p =0.633).

**Figure 1:**
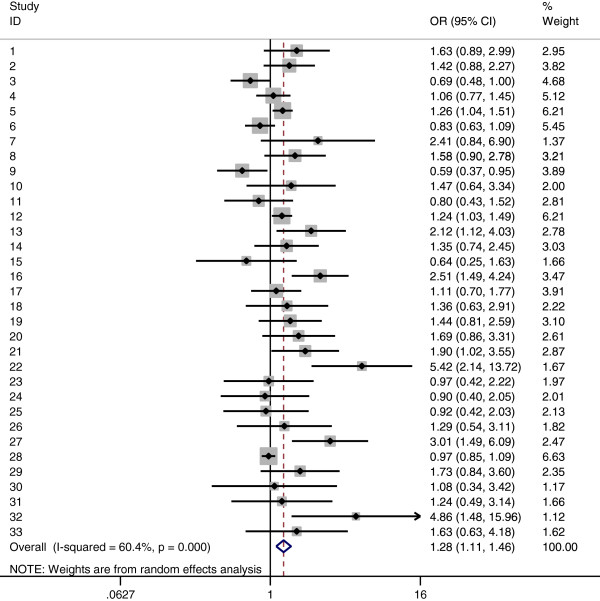
**Forest plot of cancer risk associated with the *MDR1 *3435C > T polymorphism (TT vs. CC, random effects).** The squares and horizontal lines correspond to the study-specific OR and 95% CI. The area of the squares reflects the study-specific weight (inverse of the variance). The diamonds represent the pooled OR and 95% CI.

Tables 2 summarizes the main results of the meta-analysis for *MDR1* 3435C > T polymorphisms. Overall, we found that individuals with T-allele in 3435C > T had a higher risk of cancer (co-dominant model TT versus CC: OR =1.286, 95% CI =1.123–1.474; CT versus CC: OR = 1.126, 95% CI = 1.020–1.244; dominant model TT + CT versus CC: OR = 1.176, 95% CI = 1.068–1.295; recessive model TT versus CT + CC: OR =1.191, 95% CI =1.065–1.333). In the subgroup analysis by cancer type, the results indicated that individuals with T-allele in 3435C > T had significantly higher ALL risks (TT versus CC: OR =1.890, 95% CI =1.177–3.037), otherwise no significant association was found between higher CRC risks and T-allele in 3435C > T, neither was between breast cancer and T-allele in 3435C > T. When stratified by ethnicity, significantly elevated risks were observed among Caucasian populations(co-dominant model TT versus CC: OR =1.276, 95% CI =1.112–1.464; CT versus CC: OR = 1.172, 95% CI = 1.047–1.313; dominant model TT + CT versus CC: OR = 1.212, 95% CI = 1.083–1.357), whereas significantly elevated risks were not observed among Asian populations (co-dominant model TT versus CC: OR =1.314, 95% CI =0.894–1.933). When restricting the analysis to the source of controls, we found that HB genetic models had higher risks (TT versus CC: OR =1.307, 95% CI =1.046–1.632; dominant model: OR =1.170, 95% CI =1.009–1.357), and association was detected in PB genetic models also (TT versus CC: OR =1.294, 95% CI =1.079–1.552; CT versus CC: OR = 1.150, 95% CI = 1.019–1.299; dominant model TT + CT versus CC: OR = 1.459, 95% CI = 1.246–1.709; recessive model TT versus CT + CC: OR =1.183, 95% CI =1.033–1.355) (Figure 1).

**Table 2:** **Stratified analysis of the *MDR1 *3435C > T polymorphism on cancer risk**

**Genetic model**		**Homozygote**			**Heterozygote**			**Dominant model**			**Recessive model**		
	**Sample size (case/control)**	**OR (95% CI)**	**p**_**h**_	**I**^**2 **^**(%)**	**OR (95% CI)**	**p**_**h**_	**I**^**2 **^**(%)**	**OR (95% CI)**	**p**_**h**_	**I**^**2 **^**(%)**	**OR (95% CI)**	**p**_**h**_	**I**^**2 **^**(%)**
		TT vs. CC			TC vs. CC			TT + TC vs. CC			TT vs. TC + CC		
C3435T	52(15789/20274)	1.286(1.123-1.474)	0.000	71.30%	1.126(1.020-1.244)	0.000	58.20%	1.176(1.068-1.295)	0.000	61.60%	1.191(1.065-1.333)	0.000	73.00%
Cancer types													
Acute lymphoblastic leukemia (ALL)	8(1381/1459)	1.890(1.177-3.037)	0.000	77.60%	0.981(0.683-1.410)	0.001	70.10%	1.240(1.046-1.470)	0.153	34.50%	1.829(1.095-3.054)	0.000	87.30%
Colorectal cancer	14(6362/7274)	1.047(0.875-1.254)	0.006	55.90%	1.053(0.906-1.224)	0.013	51.80%	1.054(0.907-1.225)	0.005	56.60%	1.014(0.939-1.094)	0.037	44.50%
breast cancer	9(5073/7498)	1.187(0.869-1.621)	0.001	69.30%	0.992(0.912-1.079)	0.190	28.70%	1.098(0.906-1.331)	0.024	54.80%	1.134(0.905-1.420)	0.017	57.10%
Ethnicity													
Caucasian	33(12146/15989)	1.276(1.112-1.464)	0.000	60.40%	1.172(1.047-1.313)	0.000	54.9%	1.212 (1.083-1.357)	0.000	60.2%	1.062 (1.005-1.122)	0.001	47.7%
Asian	17(2966/3418)	1.314(0.894-1.933)	0.000	80.4%	1.072(0.837-1.374)	0.000	67.4%	1.153(0.915-1.453)	0.000	67.9%	1.236(0.867-1.763)	0.000	84.4%
Source of control													
HB	25(5553/6173)	1.307(1.046-1.632)	0.000	69.10%	1.090(0.913-1.302)	0.000	64.60%	1.170(1.009-1.357)	0.001	54.90%	1.221(0.995-1.498)	0.000	76.80%
PB	26(10143/13992)	1.294(1.079-1.552)	0.000	73.90%	1.150(1.019-1.299)	0.001	53.20%	1.479(1.267-1.726)	0.000	77.60%	1.183(1.033-1.355)	0.000	69.60%
		TT vs. CC			TC vs. CC			TT + TC vs. CC			TT vs. TC + CC		
C1236T	10(2101/2842)	1.325(0.824-2.133)	0.000	77.10%	1.133(0.817-1.573)	0.005	62.20%	1.173(0.825-1.668)	0.000	70.60%	1.191(0.840-1.690)	0.000	77.80%
		TT + AA + TA vs. GG			TG + TA vs. GG			TT + AA + TA + TG + TA vs. GG			TT + AA + TA vs. TG + TA + GG		
G2677T/A	18(3585/4351)	1.348(1.031-1.762)	0.000	68.70%	1.096(0.986-1.218)	0.136	27.50%	1.161(1.051-1.281)	0.019	45.50%	1.278(1.022-1.597)	0.000	68.00%
Ethnicity													
Caucasian	11(1728/1950)	1.363(0.921-2.016)	0.000	73.60%	1.081(0.852-1.373)	0.029	50.10%	1.149(0.899-1.467)	0.007	58.80%	1.271(0.906-1.782)	0.000	74.60%
Asian	6(1583/2040)	1.642(1.340-2.012)	0.142	39.50%	1.153(0.989-1.344)	0.804	0.00%	1.273(1.101-1.471)	0.643	0.00%	1.481(1.244-1.763)	0.192	32.50%
Source of control													
HB	10(1489/2220)	1.036(0.847-1.267)	0.045	47.80%	1.025(0.874-1.202)	0.072	43.00%	1.032(0.888-1.198)	0.077	42.10%	1.028(0.867-1.219)	0.137	33.90%
PB	7(2005/2022)	1.641(1.018-2.646)	0.000	77.20%	1.164(1.008-1.345)	0.418	0.70%	1.284(1.122-1.470)	0.099	43.80%	1.537(0.998-2.366)	0.000	80.10%

*HB*: hospital-based; *PB*: population-based.

We also found that T/A-allele frequency of 2677G > T/A polymorphism was associated with higher risk of bearing cancer (2677G > T/A: TT + TA + AA vs. GG: OR =1.348, 95% CI =1.031–1.762; dominant model: OR = 1.161, 95% CI = 1.051–1.281; recessive model: OR = 1.278, 95% CI =1.022–1.597). In the subgroup analysis by ethnicity, significantly elevated risks were observed among Asian populations (co-dominant model TT + TA + AA vs. GG: OR =1.642, 95% CI =1.340–2.012; dominant model: OR = 1.273, 95% CI = 1.101–1.471; recessive model: OR = 1.481, 95% CI = 1.244–1.763), however no significantly elevated risks were observed among Caucasian populations. When stratified by the source of controls, we couldn’t find association between HB genetic models and higher cancer risks, but elevated risks could be associated with PB models (TT + TA + AA vs. GG: OR =1.641, 95% CI =1.018–2.646; GA + GT vs. GG: OR =1.164, 95% CI =1.008–1.345; dominant model: OR = 1.284, 95% CI =1.122–1.470).

The combined result based on all studies showed that there was no statistically significant link between cancer risk and 1236C > T (1236C > T: TT vs. CC: OR =1.325, 95% CI =0.824–2.133; CT vs. CC: OR = 1.133, 95% CI = 0.817–1.573; dominant model: OR = 1.173, 95% CI = 0.825–1.688; recessive model: OR = 1.191, 95% CI =0.840–1.690) (Table 2).

### Heterogeneity and sensitivity analyses

For 3435C > T polymorphism, there was substantial heterogeneity among these studies for homozygote comparison (TT vs. CC: p heterogeneity = 0.000), and recessive model comparison (TT vs. CT + CC: p heterogeneity < 0.001). Then, we assessed the source of heterogeneity for homozygote comparison (TT vs. CC) by ethnicity, cancer type and source of controls. We found that cancer type (*χ*2 = 18.51, df = 2 and p < 0.001), ethnicity (*χ*2 = 9.58, df = 1 and p = 0.002) and the source of controls (*χ*2 = 4.42, df = 1 and p = 0.036) all contributed to substantial heterogeneity. Although the sample size for cases and controls in 52 studies ranged from 38 to 5,486, the corresponding pooled ORs were not qualitatively altered with or without the study of small sample. Similarly, no other single study influenced the pooled OR materially as indicated by sensitivity analysis.

And for 2677G > T/A polymorphism, there was also heterogeneity for homozygote comparison (TT + TA + AA vs. GG: p heterogeneity = 0.000), recessive model comparison (p heterogeneity < 0.001). The heterogeneity we decided to analyze was homozygote comparison (TT + TA + AA vs. GG). Due to cancer type (*χ*2 = 20.14, df = 7 and p = 0.005) and the source of controls (*χ*2 = 14.53, df = 1 and p < 0.001), but not the ethnicity (*χ*2 = 1.28, df = 1 and p = 0.258).

### Publication bias

**Figure 2:**
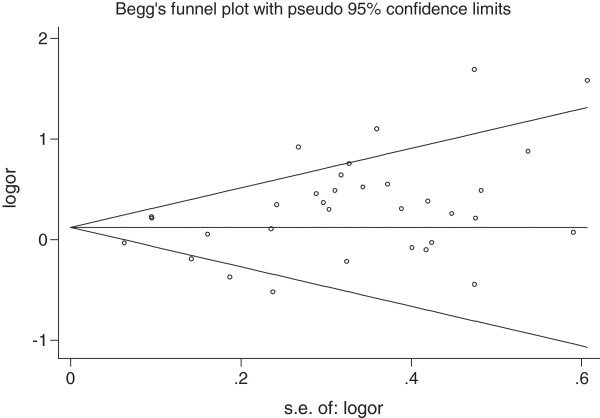
**Begg’s funnel plots for publication bias test (3435C > T TT vs. CC).** Each point represents a separate study for the indicated association. Log (OR), natural logarithm of OR. Horizontal line, means effect size.

We performed Begg’s funnel plot and Egger’s test to assess the publication bias of literatures. As shown in Figure 2, the shape of the funnel plots did not reveal any evidence of obvious asymmetry. The results of Egger’s test still did not suggest any evidence of publication bias for 3435C > T polymorphism (p =0.085 for TT vs. CC, p =0.273 for CT vs. CC, p = 0.102 for dominant model, respectively and p = 0.176 for recessive model). There are neither Publication bias for 1236C > T polymorphism (p =0.247 for TT vs. CC, p =0.208 for CT vs. CC, p = 0.215 for dominant model, respectively and p = 0.332 for recessive model), nor for 2677G > T/A polymorphism (p =0.716 for TT + TA + AA vs. GG and p = 0.656 for recessive model, p =0.841 for GT + GA vs. GG, p = 0.971 for dominant model, respectively).

## Discussion

ATP-binding cassette (ABC) is one of the largest families of active importers and exporters that are located mainly in tissues acting as a barrier or having an excretory function. Most of the ABC transporters play a role in cell defense against environmental attacks generated by xenobiotics and intraceullar metabolic waste. The multidrug resistance 1 *(MDR1/ABCB1*) gene codes for P-glycoprotein, a membrane-bound transporter. Various cytokines, such as interleukin-1beta, benzo [a]pyrene and chemokines involved in inflammation seem to be the substrates of P-glycoprotein, which leading P-glycoprotein to be a potential cause of inflammation-induced carcinogenesis. *MDR1* also seems to play a role in preventing early apoptosis in tumor cells.

Significant *MDR1* gene heterogeneity, namely multiple mutations in the *ABCB1/MDR1* gene has been demonstrated in previous studies. Analysis of all 28 exons of the ABCB1/*MDR1* gene demonstrated at least 48 single-nucleotide polymorphisms (SNPs) to date, including promoter and the intron–exon region. The most frequent SNP *MDR1* 2677G > T/A in exon 21 (RefSNP ID: rs2032582), leads to amino acid exchange from Ala to Ser or Thr. The silent mutation in exon 26 *MDR1* 3435C > T (RefSNP ID: rs1045642) is associated with altered protein function. The third common polymorphism of *ABCB1/MDR1* gene is a silent mutation in exon 12 *MDR1* 1236C > T (RefSNP ID: rs1128503). These three polymorphisms are closely related to linkage disequilibrium (LD). It was suggested that SNP is connected with susceptibility to many cancer types, such as renal epithelial tumors and acute lymphoblastic leukemia and CRC.

Our results showed that *MDR1* 3435C > T polymorphism is associated with cancer risk when all studies were pooled together (TT versus CC: OR =1.286, 95% CI =1.123–1.474; CT versus CC: OR = 1.126, 95% CI = 1.020–1.244; dominant model TT + CT versus CC: OR = 1.176, 95% CI = 1.068–1.295; recessive model TT versus CT + CC: OR =1.191, 95% CI =1.065–1.333). In the stratified analysis by cancer type, the results indicated that individuals with T-allele in 3435C > T had significantly higher ALL risks (TT versus CC: OR =1.890, 95% CI =1.177–3.037; TT + CT versus CC: OR = 1.240, 95% CI = 1.046–1.470; recessive model TT versus CT + CC: OR = 1.829, 95% CI =1.095–3.054), otherwise no significant association was found between higher CRC risks and T-allele in 3435C > T, neither was between breast cancer and T-allele in 3435C > T. When stratified by ethnicity, significantly elevated risks were observed among Caucasian populations(co-dominant model TT versus CC: OR =1.276, 95% CI =1.112–1.464; CT versus CC: OR = 1.172, 95% CI = 1.047–1.313; dominant model TT + CT versus CC: OR = 1.212, 95% CI = 1.083–1.357), whereas significantly elevated risks were not observed among Asian populations (co-dominant model TT versus CC: OR =1.314, 95% CI =0.894–1.933). When restricting the analysis to the source of controls, we found that HB genetic models had higher risks (TT versus CC: OR =1.307, 95% CI =1.046–1.632; dominant model: OR =1.170, 95% CI =1.009–1.357), and association was detected in PB genetic models also (TT versus CC: OR =1.294, 95% CI =1.079–1.552; CT versus CC: OR = 1.150, 95% CI = 1.019–1.299; dominant model TT + CT versus CC: OR = 1.459, 95% CI = 1.246–1.709; recessive model TT versus CT + CC: OR =1.183, 95% CI =1.033–1.355).

Inconsistent results might be attributed to the different roles *MDR1* played in different cell types or tissues. We’ve found the association between *MDR1* 3435C > T polymorphism and ALL risk in subgroup analyses, as well as subgroup based on HB and PB genetic models. And in the stratified analysis by ethnicity, individuals carrying the T-allele in 3435C > T were significantly associated with elevated cancer risk in Asian as well as Caucasian populations compared with C-allele carriers.

We also found that T/A-allele frequency of 2677G > T/A polymorphism was associated with higher risk of cancer (2677G > T/A: TT + TA + AA vs. GG: OR =1.348, 95% CI =1.031–1.762). In the subgroup analysis by ethnicity, significantly elevated risks were observed among Asian populations (co-dominant model TT + TA + AA vs. GG: OR =1.642, 95% CI =1.340–2.012), however no significantly elevated risks were observed among Caucasian populations. When stratified by the source of controls, we couldn’t find association between HB genetic models and higher cancer risks, but elevated risks could be associated with PB models (TT + TA + AA vs. GG: OR =1.641, 95% CI =1.018–2.646; GA + GT vs. GG: OR =1.164, 95% CI =1.008–1.345; dominant model: OR = 1.284, 95% CI =1.122–1.470).

The combined result based on all studies showed that there was no statistically significant link between cancer risk and 1236C > T (1236C > T: TT vs. CC: OR =1.325, 95% CI =0.824–2.133; CT vs. CC: OR = 1.133, 95% CI = 0.817–1.573; dominant model: OR = 1.173, 95% CI = 0.825–1.688; recessive model: OR = 1.191, 95% CI =0.840–1.690).

It should be considered that the apparent inconsistency of these results may be caused by differences in disease prevalence, lifestyle, as well as possible limitations due to the relatively small sample size. The current knowledge of carcinogenesis indicates it is a process developed step by step, as well as influenced by multiple factors that involve various genetic alterations and several signaling pathways. Thus, it is unlikely that risk factors of cancer work in isolation from each other. Besides, even the same polymorphisms may act distinct roles in each cancer type, for different genetic backgrounds may contribute to the cancer discrepancy. And more importantly, the appearance determined by polymorphisms may largely depend on synthetically interaction with each polymorphism or a particular environmental exposure. Thus, it is possible that the effects of DNA repair function on cancer risk may be modified by multiple genetic polymorphisms. Also should we consider the chance findings as another plausible reason for the inconsistency of the results.

Although we have put considerable resources and efforts into discovering the association between *MDR1* polymorphism and cancer risk as possible as we can, there still exists some limitations. First, when stratified by the source of controls, our results indicated that studies using hospital-based controls rather than population-based controls had a significantly increased risk. The reason may be that the hospital-based studies have some biases because such controls may contain certain other diverse diseases which can cause different risks of developing into cancer of various organs and may not be so representative as the general population. Therefore, using proper and more representative cancer-free control subjects are crucial for reducing biases in such case–control studies. Second, our results were based on single-factor estimates without adjustment for other risk factors such as age, smoking and drinking status, environmental factors and other variables, which might have caused serious confounding bias. Third, some inevitable publication bias might exist. Finally, the number of the published studies was not sufficiently large for a comprehensive analysis, particularly for the 1236C > T polymorphism. Hence we had to give up subgroup analysis for the polymorphism. For these limitations, our results should be interpreted with caution.

Our meta-analysis also has several strengths. First, statistically, a systematic review of the association of *MDR1* polymorphism between cancer risks is more powerful than any single study. Second, the quality of eligible studies included in current meta-analysis was satisfactory and met our inclusion criterion. Third, we did not detect sufficient publication bias indicating that the whole results might be unbiased.

In conclusion, our meta-analysis suggests that the *MDR1* 3435C > T and 2677G > T/A polymorphism may contribute to genetic susceptibility of cancers. And the results support that the minor T-allele of the *MDR1* 3435C > T polymorphism is associated with a higher risk of acute lymphocytic leukemia, and significantly elevated risks were observed among Asian and Caucasian populations as well as HB and PB subgroups. And in the 2677G > T/A polymorphism, those who carry the T-allele and A-allele were associated with higher cancer risks among Asians and PB subgroup. However, it is necessary to conduct large sample studies using standardized unbiased genotyping methods, homogeneous cancer patients and well-matched controls. Moreover, further studies estimating the effect of gene–gene and gene–environment interactions may eventually lead to our better, comprehensive understanding of the association between the *MDR1* polymorphism and cancer risk.

## Abbreviations

95% CI: 95% confidence interval; OR: Odds ratio; ALL: Acute lymphoblastic leukemia; HL: Hodgkin lymphoma; HB: Hospital-based; PB: Population-based; PCR-RFLP: Polymerase chain reaction-restriction fragment length polymorphism; MAF: Minor allele frequency; APEX: Arrayed primer extensions.

## Competing interests

The author declares that there are no competing interests and that this work has not been published or submitted concurrently for publication elsewhere.

## Authors’ contributions

LHW contributed solely to the writing and submission of this work. All authors read and approved the final manuscript.
